# Non-invasive in-vivo 3-D imaging of small animals using spatially filtered enhanced truncated-correlation photothermal coherence tomography

**DOI:** 10.1038/s41598-020-70815-3

**Published:** 2020-08-13

**Authors:** Pantea Tavakolian, Sohrab Roointan, Andreas Mandelis

**Affiliations:** Department of Mechanical and Industrial Engineering, Center for Advanced Diffusion-Wave and Photoacoustic Technologies (CADIPT), University of Toronto, Toronto, M5S 3G8 Canada

**Keywords:** Optical techniques, Imaging and sensing, Cancer imaging, Biomedical engineering, Applied physics

## Abstract

We present enhanced truncated-correlation phototothermal coherence tomography (eTC-PCT) for non-invasive three-dimensional imaging of small animals. Tumor detection is reported in a mouse thigh by injecting cancerous cells in the thigh followed by eTC-PCT imaging. Detection of the tumor 3 days after injection may lead to potential for using the eTC-PCT method for cancer treatment studies. eTC-PCT was also applied successfully to non-invasive in-vivo mouse brain structural imaging. A unique spatial-gradient-gate adaptive filter was introduced in a scanned mode along the (x,y) coordinates of camera images from different sub-cranial depths, revealing absorber true spatial extent from diffusive photothermal images and restoring pre-diffusion lateral image resolution beyond the Rayleigh criterion limit in diffusion-wave imaging science. The spatial resolution and contrast enhancement demonstrated in photothermal in-vivo and ex-vivo images of the mouse brain revealed not only vascular structures but also other brain structures, such as the brain hemispheres, cerebellum, and olfactory lobes.

## Introduction

Optical imaging provides intrinsic advantages in biological tisssue characterization through the capacity for high image contrast and resolution. However, purely optical methods are hindered by their penetration depth being effectively limited to the optical diffusion length. Recently, two-photon microscopy^1^, three-photon microscopy^2^, and optical coherence tomography^3^ were utilized for in-vivo brain imaging with the depth range of ~ 1.6 mm within the cortex layer and resolution of a few micrometers. This, however, was only possible in an invasive mode, after removing the skin and removing/thinning the skull. Although the image resolution of purely optical imaging methods is very high, the invasive nature of these methods limit their application for e.g. drug testing.

Recently, photoacoustic tomography (PAT) has been explored for non-invasive cancer tumor imaging^4–7^ and brain imaging applications as an alternative to purely optical imaging^8–13^. Through exploiting the optical-to-ultrasonic energy conversion, photoacoustic methods combine high optical contrast with the superior penetration depth of ultrasonic waves. However, the reported setups require animal models to be surrounded by a coupling medium, often water, thus limiting the potential use of PAT. They also require the use of single transducer or array scanning which complicates the instrumentation and the image acquisition process.

Light absorption and nonradiative energy conversion in the sample leads to the photothermal effect, which gives rise to thermophotonic imaging, an emerging photothermal diagnostic modality. It involves the detection of photothermal waves through emitted thermal infrared (IR) photons (Planck radiation) from tissues, captured by a mid-IR (MIR) camera. Enhanced Truncated-Correlation Photothermal Coherence Tomography (eTC-PCT)^14^ is a novel imaging method based on the photothermal effect, which has achieved superior penetration depth (4 mm in a steel sample) and axial resolution in industrial materials compared to conventional thermal imaging technologies. It also achieves a maximum lateral resolution of 16 μm, which is twice as fine as the original TC-PCT^15^ imaging. Unlike TC-PCT, the enhanced version employs a highly optimized algorithm for 3-D reconstruction, which is discussed in the supplementary section.

Here, we introduce a unique spatial gradient-window adaptive filter, revealing absorber true spatial extent from diffusive photothermal images and restoring pre-diffusion in-plane (lateral) image resolution beyond the Rayleigh criterion limitation in diffusion imaging science. Unlike PAT, this method does not require a coupling medium, thereby increasing its utility scenarios by simplifying the imaging setup and sample preparation procedures. Furthermore, it does not require scanning a linear transducer array, as the MIR camera pixel matrix is already 2-dimensional by design; and lateral spatial resolution is adaptive and adjustable, as MIR camera optics can be used to focus some or all available pixels on specific regions of interest (ROI) on the sample. In this work, using eTC-PCT with the aforementioned spatial-resolution and contrast enhancing adaptive filter, we have demonstrated the capability of this method to detect cancerous tumor growth in a mouse 3 days after cancer cell injection, as well as non-invasive non-contact structural imaging of a mouse brain. Potential impact of this work includes non-invasive monitoring of tumor drug activity and functional in-vivo imaging of drug effects in the brain of small animals. It also includes the possibility of early diagnosis and surgical guidance imaging of near-surface human tumors such as melanomas and non-melanoma skin cancers, as well as for minimally invasive endoscopy in cardiology (aorta and coronary artery wall vulnerable tissue imaging).

## Results

### Early tumor detection

**Figure 1:**
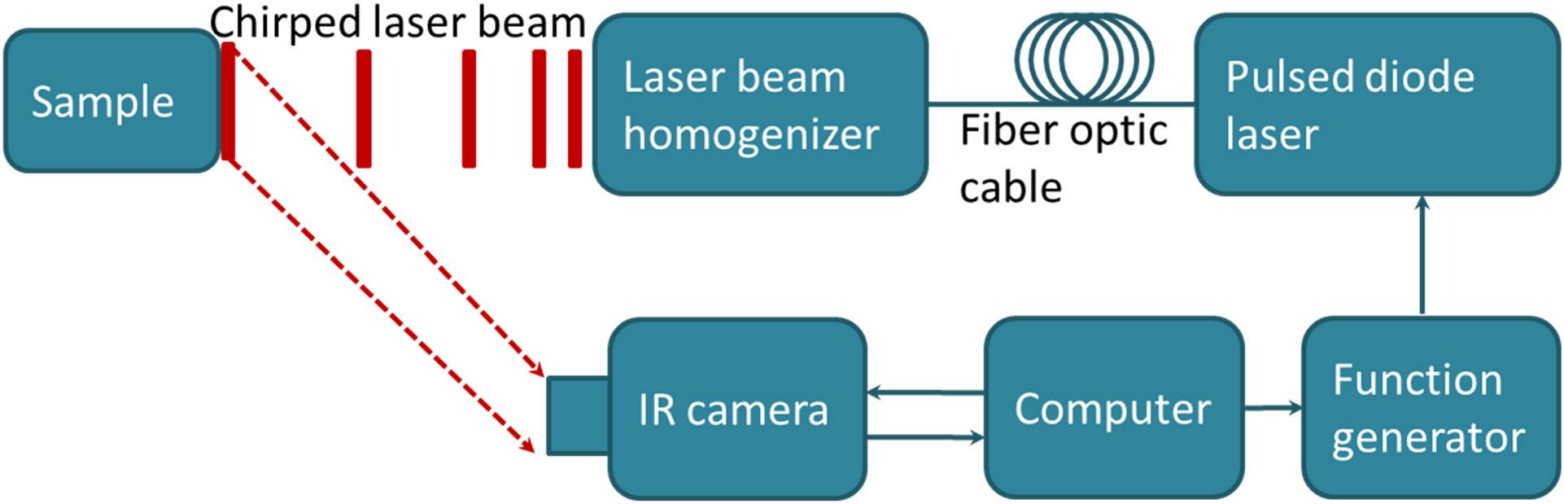
eTC-PCT system configuration.

**Figure 2:**
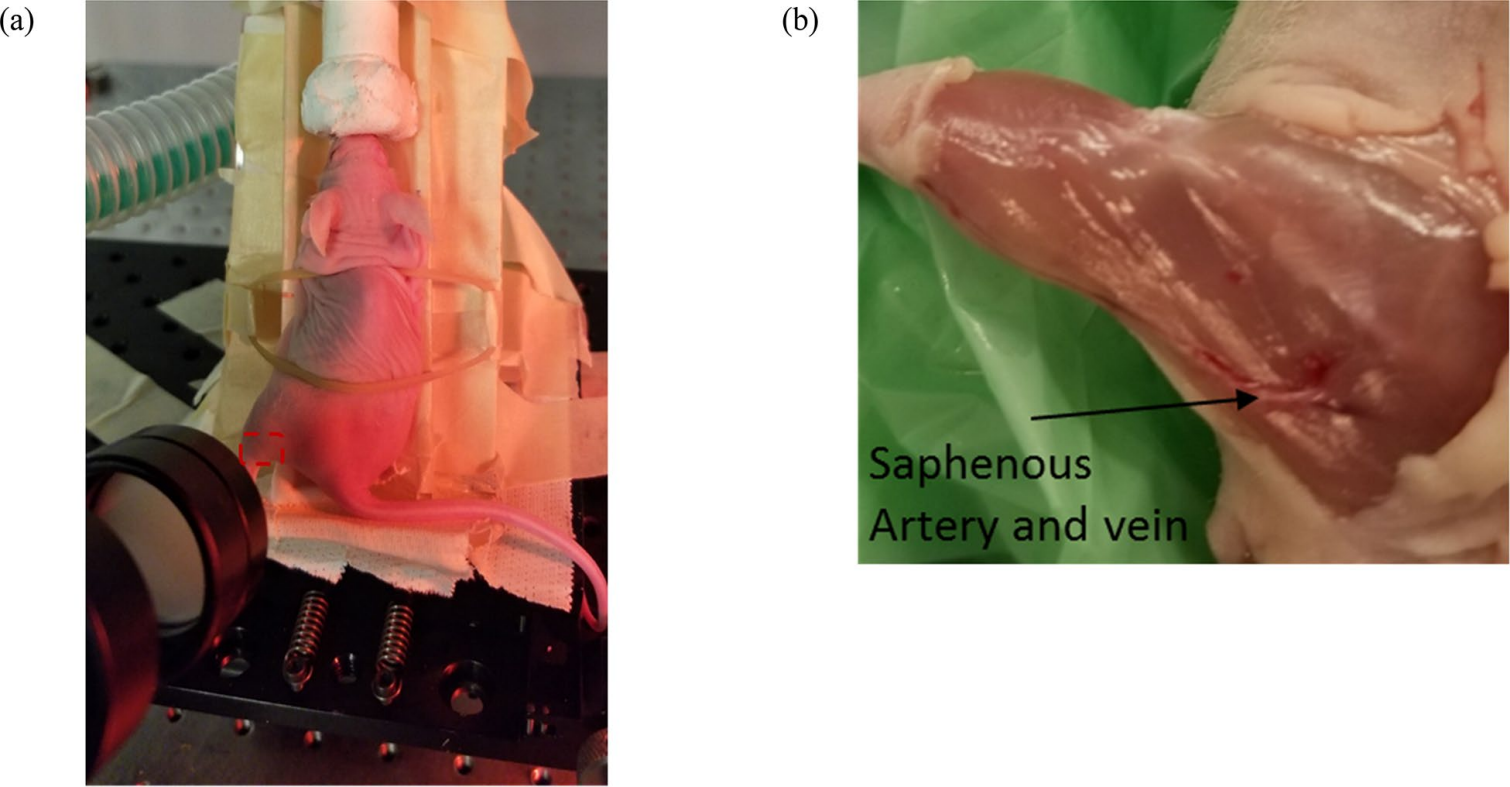
Tumor growth imaging in a live mouse. (**a**) Picture of a sleeping mouse under an anesthetic machine. The red contour shows the imaging area on the mouse thigh. (**b**) Picture of the euthanized mouse thigh with the skin removed. The saphenous artery and vein can be seen in this picture and are detected in eTC-PCT images.

The TC-PCT imaging set-up is shown in Fig. 1. A photograph of the position of a mouse placed on the holder, normal to the mid-IR camera and at its focal point, is provided in Fig. 2a with a red contour on the mouse thigh that shows the imaging ROI. The camera records the thermal evolution of the surface tissue following exposure to laser ‘chirp-1’ illumination (“Methods”). Imaging was performed before the injection of the cancer cells, and subsequently, 3 and 9 days after the injection. Afterwards, the mouse was euthanized and the leg skin was removed (Fig. 2b). The tumorous tissue was then collected and sent to Princess Margaret Hospital, Toronto, ON, for histopathology analysis.

**Figure 3:**
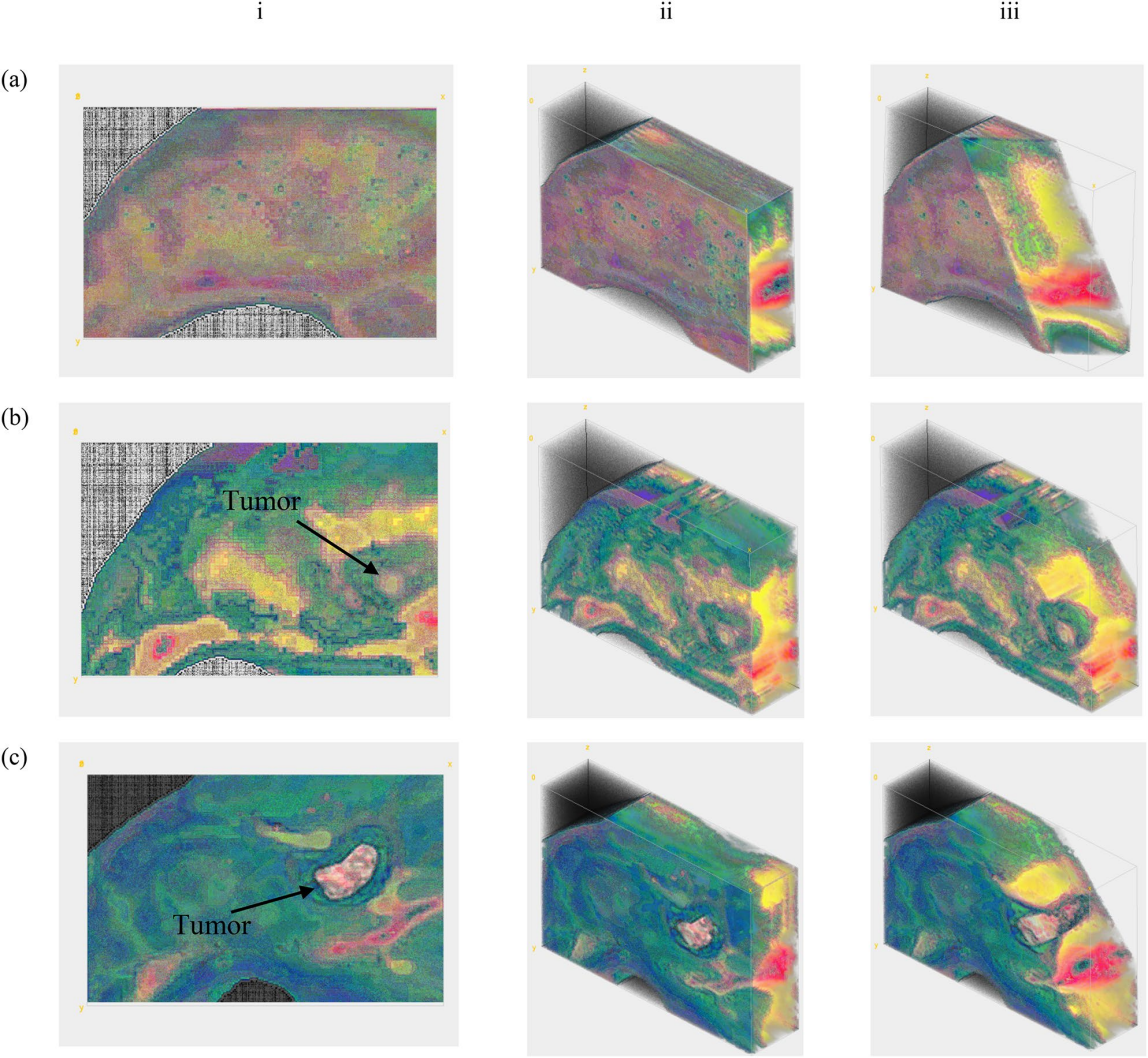
3-D eTC-PCT imaging of a tumor. (**a**) Amplitude image of the mouse thigh before injection of cancer cells. **{2.1}** (**b**) Amplitude image of the mouse thigh 3 days after injection of the cancer cells. (**c**) Amplitude image of the mouse thigh 9 days after injection of the cancerous cells. Column (i) displays an x–y view of the 3-D images; column (ii) shows a tomographic 3-D view of the thigh, and column (iii) shows a tomographic 3-D view of the thigh with the right corner of the image computationally removed to reveal the penetration depth of the tumor in the tissue. On day 3 tumor size is much smaller than on day 9. The size of each image is 1.35 cm × 1.08 cm, and **{1.4}** the depth scale is approximately 1.7 mm based on Eq. 1. These images were captured with a chirp-1 waveform.

In eTC-PCT, two cross-correlation contrast channels were generated and used for the current study: amplitude peak, and phase (“Methods”). 3-D results based on the amplitude channel can be seen in Fig. 3 for the mouse thigh before and after injection. The tissue change can be seen with eTC-PCT by the third day after injecting the cancer cells. The tomographic cross-sectional depth profile details of the tissue are seen in Fig. 3. Images taken on day 3 (Fig. 3b) clearly show that the tissue structure of the thigh has changed: The tumor has been building its own vascular network to provide oxygen and nutritional support to the affected cells, therefore, more blood vessels are generated and observed in the middle area of the thigh, Fig. 3b, where the tumor is developing. More detail becomes apparent on day 9 (Fig. 3c), where the presence of a palpable mass is evident with further growth and deep subsurface penetration of the tumor. Furthermore, the tumor has higher density than muscular tissues, which changes the thermal properties of the tissue and generates additional contrast (“contrast amplification”) beyond that due to optical property changes in the thermophotonic image.

**Figure 4:**
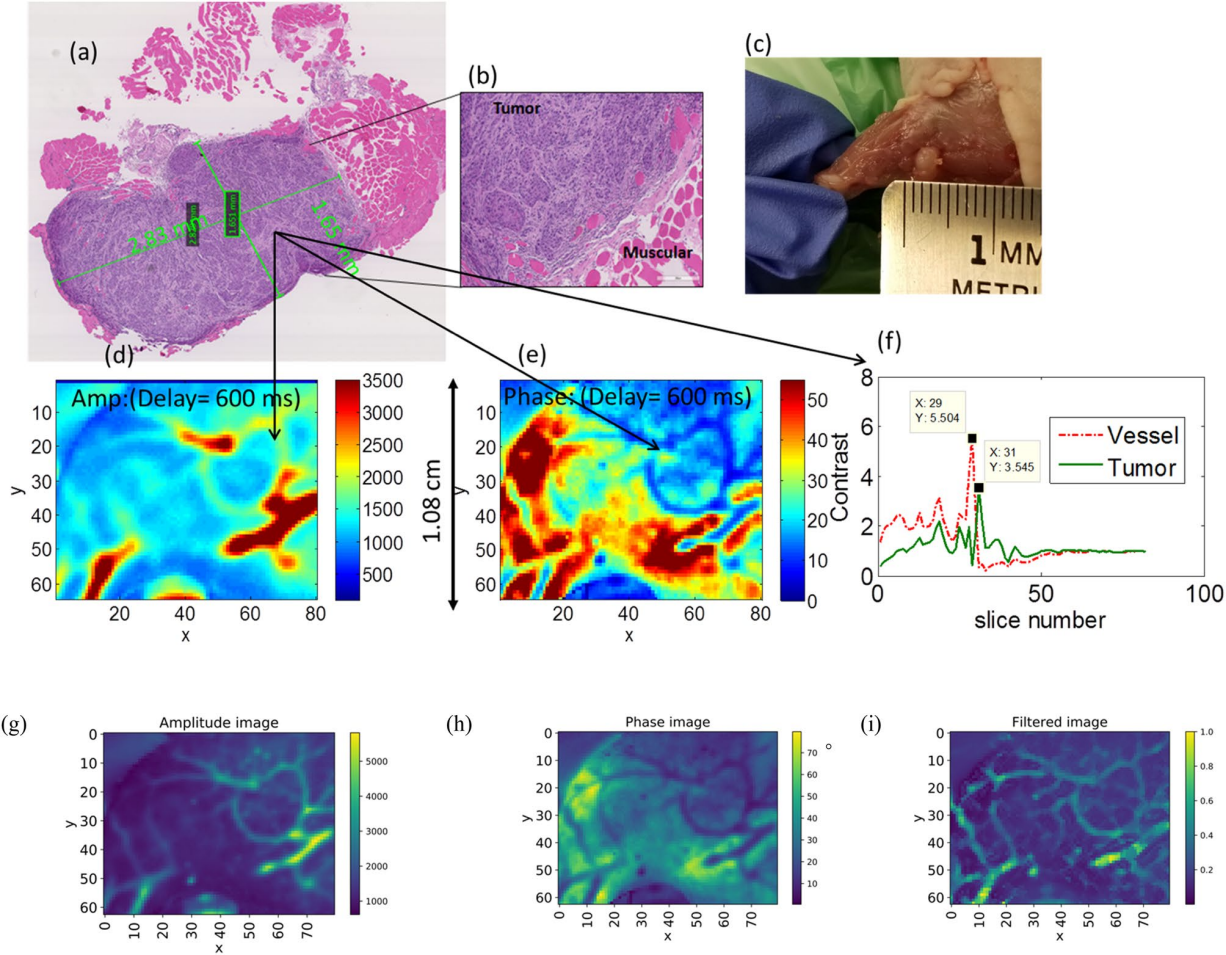
Histopathological validation. (**a**,**b**) H&E images of the collected tumor tissues. The two green quadrangles in the middle of the tumor indicate the in-software measured sizes. (**c**) Photograph of the tumorous area. (**d**) Amplitude and (**e**) Phase eTC-PCT images nine days after injection with time delay of 600 ms. (**f**) Contrast measurement for blood vessel (around the tumor) and the affected region as a function of slide number (along the depth direction). eTC-PCT amplitude (**g**), and phase (**h**) images at 400 ms. The filtered image (**i**) of combined amplitude (**g**) and phase (**h**) shows more details such as revealing the presence of smaller vessels. The color bars on the filtered image do not have units as it is derived from mixing amplitude and phase.

To confirm the presence of the tumor in the thigh of the mouse, histological validation was performed. The removed tumor tissue was viewed under a microscope. The tumor size and shape could be identified and were measured from the images. While the tumorous tissue reveals a high density of cancer cells that possess dark nuclei, normal tissue is seen as the pink sections, Fig. 4a,b. Additionally, the excised tumor sample geometry exhibited excellent correspondence with the visible-light photograph after euthanasia, Fig. 4c. The shape of tumor structure in the eTC-PCT phase and amplitude images, Fig. 4d,e, is consistent with both the histology image and the visible photograph of the excised tumor, Fig. 4a,c. Single cross-sectional image slices from each of eTC-PCT amplitude and phase results are also presented at a delay time of 600 ms in Fig. 4d,e, matching Fig. 4c well.

Based on Eq. (1) that relates time delay to depth in eTC-PCT system for an opaque sample, the approximate depths of the tumor (front surface of the tumor which first appears in the thermal image) and its surrounding vessels were estimated. These are, however, not accurate measurements due to the turbid nature of biological tissue which results in (photo)thermal sources at different depths based on the optical field generated from the coherent and the scattered laser beam components, leading to diffusive propagation^16^.

1 $$z=\frac{2\times \sqrt{\alpha t}}{\sqrt{\pi }}$$

Assuming the thermal diffusivity (α) of tissue to be 2.72 × 10^–3^ cm^2^.s^−1^^17^, Eq. 1 predicts that the tumorous region exhibited its maximum contrast in the eTC-PCT tomographic image at the depth of 1 mm, Fig. 4f. The vascular network around the tumor exhibited its highest contrast closer to the skin (slice 29) than the tumor (slice 31) with 65 µm difference. The real depth of the tumor on Day 9 was measured to be 1.5 to 2 mm beneath the skin; the tumor was 0.5-mm thick. The smaller depth calculated from Eq. (1) is consistent with the validity of that equation for surface-absorbing opaque media where deeper thermal-wave generation following subsurface optical penetration is absent.

To enhance the eTC-PCT image contrast and resolution, a spatial gradient-window adaptive filter for minimizing the effect of thermal diffusion-wave broadening and signal overlapping along the *x* and *y* coordinates was developed (“Methods”). Side by side amplitude and phase images of the thigh on day 9 along with the filtered image is presented in Fig. 4g–i. Spatial resolution estimated in the filtered image is 168 μm.

### Structural brain imaging

Photothermal imaging of the brains of two live mice was achieved noninvasively with the skin and skull intact. The mice were placed under anesthesia as the first step and for the duration of each experiment.

(a) followed by eTC-PCT imaging of the brain. Then, each mouse was euthanized and further imaging of the brain was performed after removal of the scalp. Open-skull visible-light photographs of the cortex vasculature of the euthanized mice,

(b) and 6(a), were produced. The combined thickness of the skin and skull of the adult mice covering the brain was measured to be ~ 0.95 mm, and ~ 0.9 mm.

To enhance the brain image contrast and resolution, a spatial gradient-window adaptive filter is applied to eTC-PCT images. The thermal response of the brain along one line of the image is plotted in blue, while the filtered signal is plotted in red,

(c) The adaptive filter was applied after mixing the amplitude and the phase eTC-PCT results (“Methods”). Brain imaging results are presented for two cases. First, in-vivo non-invasive images were captured when the mouse was asleep under the anesthetic machine,

(d) Next, images in the absence of the skin were captured after the mouse was euthanized,

(e) These cross-sectional images correspond to images of the superficial cortex (starting at 0 ms) and interior brain structures (up to 3.45 s) from the dorsal to the ventral part of the brain. These images are captured with the ‘chirp-2’ waveform (“Methods”).

In the process of removing the skull of the first mouse, the cutter accidentally touched the left hemisphere of the brain, which resulted in an excess of blood on the brain surface as can be seen in the photograph of the mouse brain imag.

(b) This however did not happen for the second mouse. The photograph of the brain along with its dc IR image is presented in Fig. 6a,b, respectively. The brain anatomy of a laboratory mouse can be seen in Fig. 6c^18^. For illustration, the eTC-PCT images of the brain are compared with (c) to examine tissue matching between them.

## Discussion

This work reports the development and optimization of the eTC-PCT method for small animal imaging. This method integrates deeper penetration than pure optical imaging, higher spatiotemporal resolution compared to other thermal imaging modalities, high sensitivity to vascular detection, and 2-D/3-D tomographic imaging capabilities. Imaging of the entire mouse thigh before and after cancer cell injection, and imaging of the entire mouse head were performed with high speed (less than 80 s). Since eTC-PCT is non-invasive and involves a relatively simple and in-expensive thermophotonic system which, unlike PAT or ultrasound imaging, does not require a coupling medium such as a fluid for impedance matching purposes, it can easily be used for preclinical cancer studies and animal drug testing.

Angiogenesis generates blood-rich regions which play a central role in cancer development and metastasis, an essential hallmark by which eTC-PCT differentiates tumors from normal tissues. Imaging on the 3rd day after injection exhibited an early-diagnostic result for the emerging thigh tumor as the images showed the formation of the vasculature surrounding the tumor on that day. The tumor was visually invisible even on day 9th.

To capture eTC-PCT images, a near-infrared laser beam was used which provides high penetration depth within biological tissue^19^. Higher penetration depth can be achieved using longer excitation wavelength^2,3^. In the case of tumor imaging, the high penetration of 808 nm enables photons to be transmitted into tissue and to be absorbed by the vascular network surrounding the tumor. eTC-PCT tomographic images are sensitive to the presence of the increased angiogenetic blood vascular network in the tumor, since blood has higher absorption coefficient than the surrounding tissue at 808 nm wavelength, leading to high contrast tumor imaging. As a result, the presence of a tumor in the core of the vasculature network can be seen in Fig. 3 on days 3 and 9. The tumor size on day 3 was very small. On day 9, the tumor size increased to 2.8 mm × 1.6 mm as measured with the histological image. The confirmed shape and size of the tumor matches well with the eTC-PCT results for day 9, Fig. 4. In Fig. 4d,e, the ring-shaped vasculature network around the bean shaped tumor is clearly visible.

Data analysis based on truncated temporal slices at different depths yields approximate depth information of a specific light absorbing tissue based on Eq. 1, as shown in Fig. 4f. In that figure, the tumor appears with highest contrast at slice 31, while one vessel has the contrast peak at slice 29. From these peaks and Eq. 1, one can approximately measure the imaged ROI’s axial depth. In this case, the tumor within the thigh was ~ 65 µm deeper than the imaged blood vessel. As expected, the vascular network appears with higher eTC-PCT image amplitude and smaller phase lag compared to muscular tissue, Fig. 4d,e. Additional thermophotonic contrast amplification occurs because the tumor has higher density and therefore smaller thermal diffusivity than the muscular tissue.

**Figure 5:**
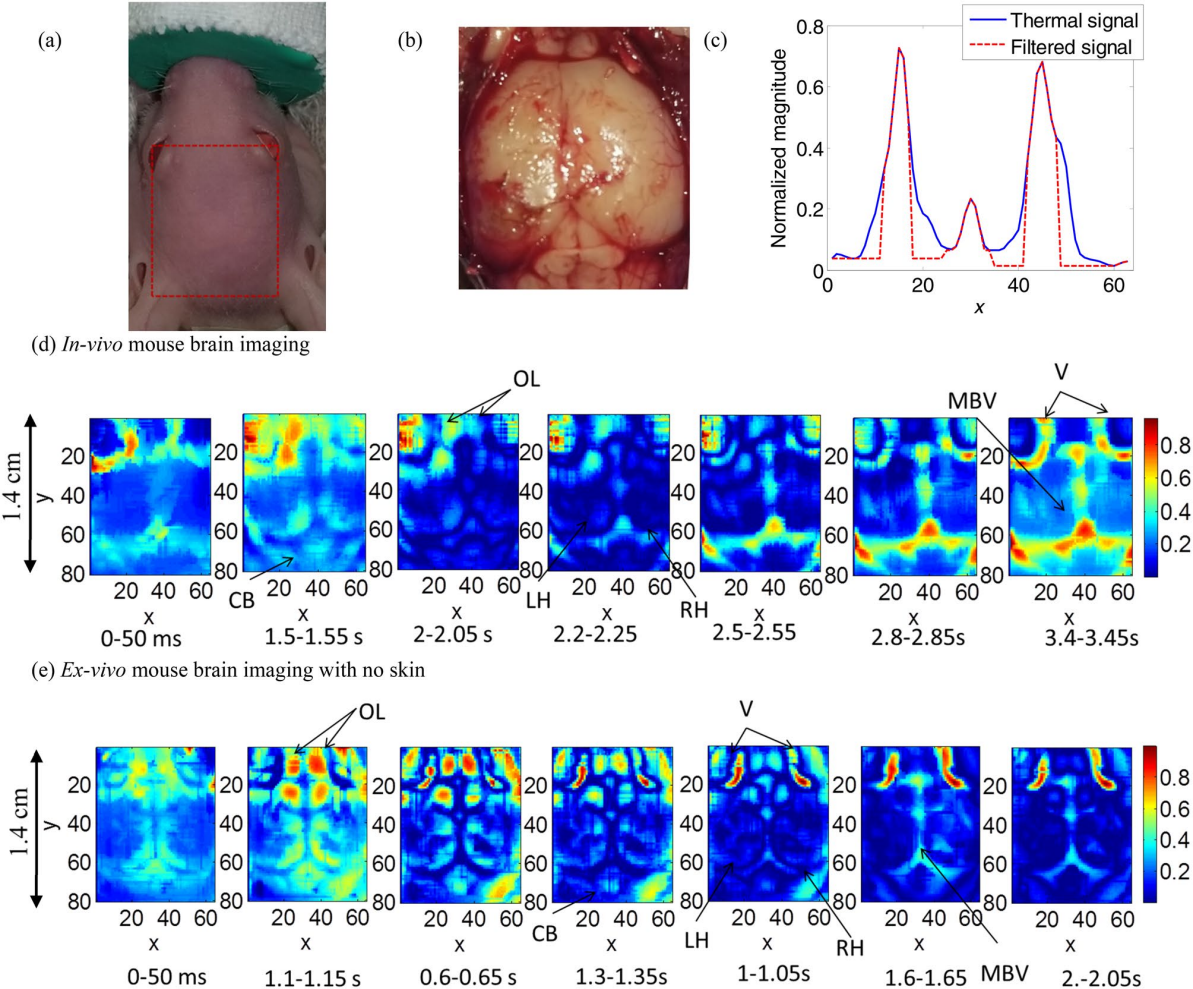
eTC-PCT imaging of a mouse brain. (**a**) Mouse picture under the anesthetic machine. Imaging area on the mouse head is shown in the red rectangle. (**b**) An ex-vivo open-skull photograph of the cortex vasculature. The bloody area on the bottom left is a result of accidental contact of scissors with a blood vessel. (**c**) Thermal signal along one line of 2-D eTC-PCT image captured by the camera at $time{=t}_{j}$ in blue with its filtered version in red. (**d**) eTC-PCT non-invasive in-vivo brain images; and (**e**) eTC-PCT brain images after removing the skin at different depths. Arrow-associated acronyms are defined in the text. The images show horizontal cross sections from the dorsal to the ventral part of the brain, where the imaging depth is increasing from the top surface of the mouse head with time. The color bars on the images do not have units as they are derived from mixing amplitude and phase. The size of each image is 1.4 cm × 1.12 cm. These images are captured with chirp-2 waveform and are resolution and contrast enhanced using the spatial gradient-window adaptive filter. The color bars on the images do not have units as they are derived from mixing amplitude and phase.

Part of the experimental results presented in this study demonstrates the first application of a thermophotonic imaging modality to live animal brain imaging. 3-D eTC-PCT was used as a non-invasive method for characterizing the local opto-thermal properties of in-vivo mouse brain tissues through the skin and skull. The completely non-invasive, non-contacting nature of this modality and without the need for fluid coupling, it has the potential to be used for full-area fast functional imaging with image acquisition time of ~ 1 min. The diffusive nature of thermal imaging degrades the eTC-PCT image resolution which is limited by a compromised Rayleigh criterion for thermal diffusion^20^ especially for subsurface imaging such as in the presence of skin and skull overlayers. The spatial gradient-window adaptive filter introduced in this work operates as a resonant gate that produces a peak when the spatial width of the window matches the steepest lateral gradient of signals produced by overlapping radial or sideways diffusion from absorber boundaries (“Methods”). As a result of filtering, measurable improvement of the tumor images in Fig. 4g,h can be observed in Fig. 4i with more details, finer and deeper vessels becoming visible with spatial resolution estimated to be 168 μm in sub-window mode with averaging of 4 pixels. More substantial resolution improvement was observed in the cases where imaging features lay beneath the dense and light scattering skull overlayer of mouse brain, Fig. 5. The skull layer causes profuse optical scattering of the incident laser beam more so than going through the soft thigh tissue; it also induces stronger lateral thermal diffusion of the back-propagating thermophotonic response.

Mixing the amplitude and the phase eTC-PCT images, and applying the adaptive filter algorithm assisted in restoring thermophotonic image resolution very close to the pre-diffusion optical resolution as shown in .

(d,e) while enhancing contrast against the background as indicated in.

(c) This constitutes a resolution breakthrough beyond the diffusion-wave Rayleigh criterion limitation.

**Figure 6:**
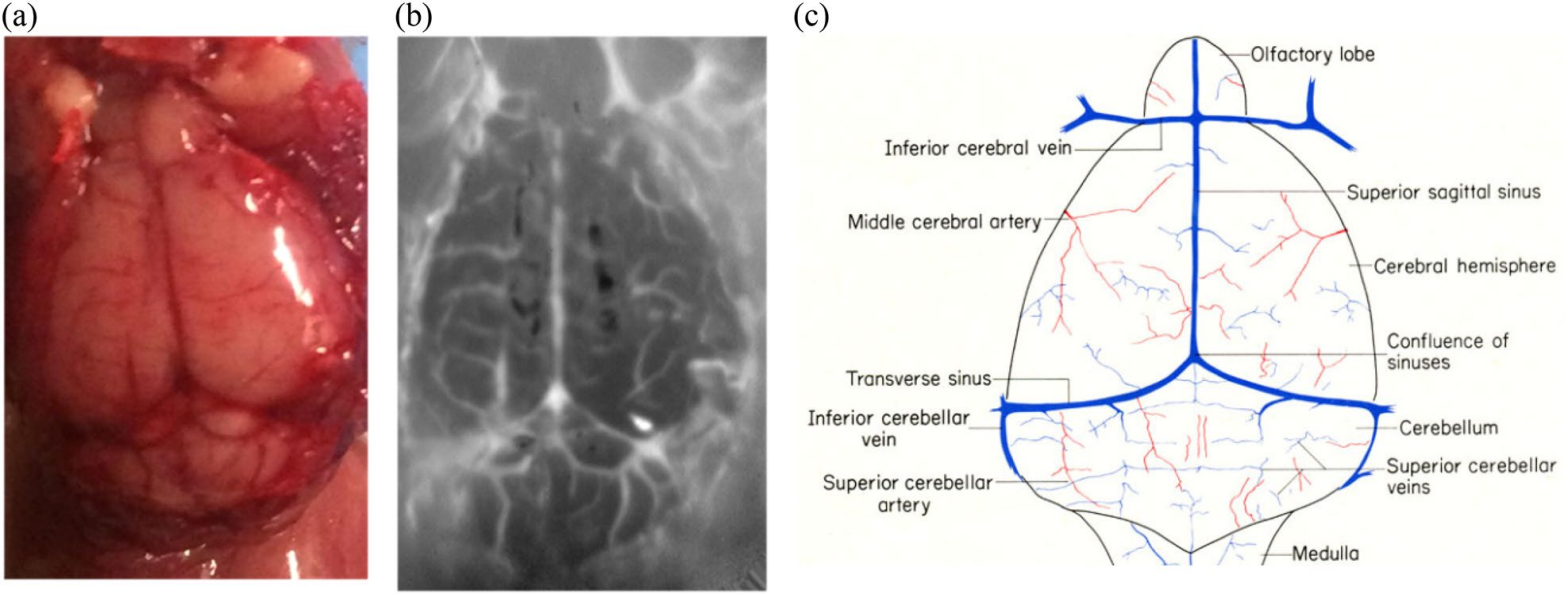
Brain structural image (**a**) Photograph of the brain image of the second mouse. (**b**) IR image of the second mouse brain. (**c**) Brain anatomy image is presented (from literature^18^) for comparison with the eTC-PCT images.

Blood vessels and vascular distribution in the brain can be seen in eTC-PCT images with high optical contrast,

(d,e) The results for all the slices are presented on the same color scale. These images show structures such as blood vessels (V), middle blood vessel (MBV), cerebellum (CB), left hemisphere (LH), olfactory lobes (OL), and right hemisphere (RH), also clearly depicted in Fig. 6c. The dc IR image of the second mouse brain surface is shown in Fig. 6b confirms the ability of the MIR camera for very high lateral spatial resolution in the absence of skin and skull layer. With skin intact the lateral resolution of 0.3 mm was achieved in sub-window mode. The resolution was measured based on the full-width at half maximum of the phase data from the thinnest vessel that was identified in the eTC-PCT phase and amplitude image. Resolution of the full-window IR image of the brain shown in Fig. 6b is 60 μm. Resolution can improve to 16 μm in the presence of the skin using the camera at its full-window functionality focused on a smaller area, without pixel averaging (“Methods”). This needs a high-speed computer with minimum 128 GB RAM for compiling the post-processing algorithm on the large amount of data recorded by the camera. An alternative method for obtaining high resolution images of the brain is to remove a small area of the mouse scalp and apply in-vivo eTC-PCT imaging as performed with other photonic and hybrid modalities^1–3^. The present imaging results in the absence of the scalp,

(e) provide more details of cerebral blood vessels compared to in-vivo images. Thinning of the skull layer is also another option which will improve the results in terms of resolution and penetration depth as has also been demonstrated with two-photon microscopy^1,21^.

In conclusion, biothermophotonic 3-D eTC-PCT was introduced and was shown to detect the presence of an early stage tumor in a mouse thigh with measurable size and shape, which is highly desirable towards early clinical diagnosis and potential use in pharmacology and pharmacokinetics. With dynamic spatial filtering, eTC-PCT imaging, very sensitive to the presence of blood, proved capable of detecting blood vessels under the skull and skin layer. The completely non-contacting advantage of eTC-PCT improves data consistency and avoids time-consuming animal preparation complexities compared with photoacoustic and ultrasonic brain imaging. eTC-PCT has a spatial resolution (300 μm in brain imaging after averaging several pixels which can be improved without averaging, and 170 μm after applying the filtering in the tumor detection) comparable with photoacoustic imaging with a resolution of 125 μm^22^. Other optical modalities^1–3^ such as photoacoustic microscopy with the resolution of a few/submicrometers, are achieved invasively. Further studies are underway using a differential eTC-PCT approach with two-wavelength beams for brain functional imaging with improved contrast.

## Materials and methods

### Experimental set-up

The experimental setup of the eTC-PCT system is shown schematically in Fig. 1. It includes an IR camera (A6700sc, FLIR, USA, 3–5 μm spectral response), which records the thermal evolution of the target sample following exposure to laser irradiation. A function generator (Keysight 33500B, USA) is used to generate a linear frequency modulation (LFM) chirp. The LFM excitation chirp signal controlls the diode laser (Jenoptic JOLD- 120-QPXF-2P) through a laser driver (PCO-6131, Directed Energy, Colorado, USA), and is recorded using a high-speed data acquisition module (NI PCI-6281) for synthesizing the reference chirp. The laser beam is passed through a collimator (F22SMA-B, Thorlabs Inc., New Jersey, USA), and a diffuser (ED1-C20, Thorlabs Inc., New Jersey, USA) to become collimated, expanded, and homogenized^14^.

A camera frame rate of 104 Hz was used for both brain and tumor imaging. The frame size of 14 mm × 11.2 mm was employed for the brain imaging experiments, while a smaller frame size (13.5 mm × 10.8 mm) was used for the tumor studies. The camera was used at sub-window mode of 320 × 256 pixels, and four neighboring pixels were averaged to produce an 80 × 64 image size. An in-house developed eTC-PCT reconstruction algorithm was utilized to create 2-D depth planar and 3-D temporally truncated cross-correlation (CC) images of the mice used in this work.

The eTC-PCT algorithm developed for biomedical imaging provides slice-by-slice cross-correlation (CC) data for the amplitude and phase channels for all camera pixels. The synthesized reference chirp is cross-correlated with the photothermal relaxation signal of that pixel (Supplementary). The CC is calculated at time intervals determined by “slice width”, *W*_*T*_, and an “incremental delay unit”, *d*, where *W*_*T*_ is controlled by the user and is used for the calculation of *d*. Next, the calculated CC is truncated by a time gating filter to provide depth resolved information. Finally, for each *d*, compilation of the data from all pixels leads to a depth-resolved “slice” image of the sample. The photothermal relaxation signals carry information about the optical and thermal properties of the layers beneath the skin surface of the mouse, with information from deeper layers taking a longer time to arrive conductively to the surface. eTC-PCT axial resolution is determined by *W*_*T*_. A longer *W*_*T*_ will compile the data from more recorded frames for CC calculation of each pixel and thus results in higher contrast and SNR, at the cost of reducing axial resolution^14^.

### Spatial-gradient-gate adaptive filter for thermophotonic image resolution restoration

Assuming a temporal Dirac delta function (spatially impulsive) absorption line in homogeneous and isotropic space, the one-dimensional temperature field along the *x* coordinate ( *x* = *0* on the surface) can be written as a diffusive function of time (*t*) and the object’s thermal diffusivity (α):

2 $${\varvec{F}}\left({\varvec{x}},{\varvec{t}}\right)=\frac{1}{\sqrt{{\varvec{\pi}}\boldsymbol{\alpha }{\varvec{t}}}}{{\varvec{e}}}^{-{{\varvec{x}}}^{2}/4\boldsymbol{\alpha }{\varvec{t}}}$$

For simplicity, the time-dependent pre-exponential factor can be ignored or lumped into **{1.13}** a constant $A_{j} \equiv A(t_{j} ) = 1/\sqrt {\pi \alpha t_{j} }$ for a fixed instant *t* = *t*_*j*_ at which a camera image is recorded. The spatial gradient-window adaptive filter consists of two scanning coordinate points ${x}_{1}$ and ${x}_{2}$ which constitute a moving spatial gate of width ∆*x* = *x*_*2*_* – x*_*1*_ and are related to each other through a constant *c* (adjustable, but fixed throughout a given scan) as follows:

3a $$x_{2} /x_{1} = c$$

As these spatial points, subject to the constraint (3a), are scanned along each *x* coordinate (pixel) line within a pre-determined *(x,y)* area of the instantaneous image recorded by the camera, the function

3b $$g(x_{1} ,x_{2} ;t_{j} ) = F(x_{1} ,t_{j} ) - F(x_{2} ,t_{j} ) = A(t_{j} )\left( {e^{{ - \left[ {x_{1} /X(t_{j} )} \right]^{2} }} - e^{{ - \left[ {x_{2} /X(t)_{j} } \right]^{2} }} } \right)$$

where

3c $$X(t_{j} ) = 2\sqrt {\alpha t_{j} }$$

is a thermal diffusion length of signals generated following optical absorption and nonradiative energy conversion to a heat source which subsequently diffuses during time *t* = *t*_*j*_ , Eq. (2). The function *g* represents a moving spatial signal contrast window of width ∆*x.* If the constant *c* that determines the fixed distance between *x*_*1*_ and *x*_*2*_:

$x_{2} - x_{1} = (c - 1)x_{1}$, and makes *g* a function of *x*_*1*_ only, is set so that *g* exhibits a maximum value under the condition

4 $$\frac{{dg(x_{1} ,t_{j} )}}{{dx_{1} }} = A_{j} (t_{j} )\frac{d}{{dx_{1} }}\left( {e^{{ - \left[ {x_{1} /X(t_{j} )} \right]^{2} }} - e^{{ - c^{2} \left[ {x_{1} /X(t_{j} )} \right]^{2} }} } \right) = 0$$

the maximum of *g* occurs within the scanned spatial window $\Delta x_{\max } = (x_{2} - x_{1} )_{\max }$ when *x*_*2*_ is related to *x*_*1*_ through the condition Eq. (4) which yields

5 $$X(t_{j} ) = \left[ {\frac{{x_{2}^{2} - x_{1}^{2} }}{{2\ln \left( {x_{2} /x_{1} } \right)}}} \right]^{1/2}$$

Manipulating Eq. (5) and using Eq. (3a) leads to the condition for the appearance of a *g(x*_*1*_*,t*_*j*_*)* maximum when the spatial gate width ∆*x* satisfies the relation

6 $$\Delta x = \Delta x_{\max } = (c - 1)X(t_{j} )\left( {\frac{2\ln c}{{c^{2} - 1}}} \right)^{1/2}$$

This relation shows that if *c* (and thus ∆*x*) is chosen appropriately, the gradient of the moving spatial window *g(x*_*1*_*,t*_*j*_*)*, Eq. (6), matches the steepest spatial gradient of the laterally diffusing signal gate, Eq. (3b), when the latter lies within the moving (scanning) spatial window ∆*x.* It is well-known that the steepest thermal gradient occurs at the effective edge of an optical absorber acting as a thermal source. The foregoing physical process represents a form of spatial resonance (matching) between the slopes of the decaying diffusive profile *g(x*_*1*_*,t*_*j*_*)* and ∆*x*_*max*_ .

In the eTC-PCT images, *g(x,t*_*j*_*)* represents the signal difference at the two locations:

7 $$g(x,t_{j} ) = S(x_{1} ,t_{j} ) - S(x_{2} ,t_{j} )$$

generated by finite-size absorbing regions in the image. In this manner, thermal-diffusion-broadened boundaries beyond absorber edges, especially at large depths, are identified following steepest decay gradients and filtered out, whence lateral image resolution is approximately restored to true absorber sizes. Therefore, the position of the *g(x,t*_*j*_*)* peak obtained from scanning the ∆*x*_*max*_ gate becomes the criterion for lateral resolution enhancement. In practice, *c* > *1* can be chosen to be small so that *x*_*1*_ and *x*_*2*_ are close when signal gradients are expected to be steep or at early diffusion times, thus enhancing image lateral spatial resolution. *c* can be chosen to be large when gradients are small or at longer diffusion times, spreading out in space, so that *x*_*1*_ and *x*_*2*_ are relatively farther apart and spatial resolution is expected to be low. The optimal value of *c* in a given image configuration is obtained (“adapted”) for maximum peak value starting the scanning when $g\left({x}_{m},t\right)$ is close to zero and lateral image cut-off is determined by *x *_*max*_* ,* the coordinate point *x*_*1*_ at which the ∆*x*_*max*_ maximum occurs. This turns out to be a good approximation. The precise location of the *g(x,t*_*j*_*)* maximum can be found from Eqs. (3b) and (5) to be at

8 $$x_{1,\max } (t_{j} ) = \left( {\frac{2\ln c}{{c^{2} - 1}}} \right)^{1/2} X(t_{j} )$$

It is noted that the position of the maximum and Eq. (3c) can be used to calculate the thermal diffusivity of the diffusing feature.

(c) displays an example of a filtered signal (in red) of the thermally broadened absorption (in blue) along the *x* coordinates (from pixel 1 to 64 at *y* = *5*, and at the depth location corresponding to *t* = 50 ms)*.* This algorithm is applied to the sequence of broadened features along both the *x* and the *y* axes leading to two filtered images. Then, the two computed images are averaged. The thermophotonic image resolution restoration filter is applied to the mixed eTC-PCT amplitude and phase images. The mixed images are obtained by multiplying the amplitude and phase results at each pixel, providing details of both channels in one.

### Cancer cell preparation

Human hypopharyngeal head and neck squamouscell carcinoma FaDu cell lines, obtained from the American Type Culture Collection (Manassas, VA), were cultured in MEM F-15 supplemented with 10% fetal bovine serum. The right thigh of the nude mouse was injected subcutaneously with 4.8 × 10^6^ cultured cells/30 μl and imaged consecutively over a two week period (results presented here are from two sessions).

### Histopathology analysis

The collected tumorous tissues were fixed in 4% of neutral-buffered formalin and routinely processed in paraffin wax. Hematoxylin and eosin (H & E) staining was conducted using standard procedures. The stained sections were reviewed under a microscope. H&E staining showed a cellular neoplasm arranged in nests and covered by fibrotic pseudocapsule. The surrounding healthy muscular tissues showed eosinophilic hyaline staining.

### Imaging procedure

For brain and tumor imaging experiments, four adult CD-1Nude mice (Charles River Breeding Laboratories) were used, two mice for each category, according to protocol 20011804 approved by the DCM of the Faculty of Medicine, University of Toronto. Animal handling was also performed according to guidelines for laboratory animal care.

In each experiment one mouse was anesthetized by administrating 1 L/min of oxygen mixed with isofluorane gas (3% for induction and 1–1.5% for maintenance). To regulate the animal temperature, a heating IR lamp was used. The animal was laid on a bed normal to the camera. The ROI was then illuminated and the thermophotonic slice/signal evolution was detected with the camera, while the mouse was sleeping under the anesthetic machine. Finally, the collected thermal signals were post processed and reconstructed with the eTC-PCT algorithm, generating in-vivo images of the mouse head or thigh.

The experimental pulsed chirp radar parameters, designated as ‘chirp-1’ waveform, were: Starting frequency 0.07 Hz, ending frequency 0.1 Hz, and chirp duration 81 s. Additionally, ‘chirp-2’ waveform parameters were: Starting frequency 0.03 Hz, ending frequency 0.1 Hz, and chirp duration 81 s. Homogenized laser beam diameter was 3 cm for tumor imaging and 3.6 cm for brain imaging. The excitation pulse width was 70 ms for tumor imaging and 140 ms for brain imaging, resulting in energy density of 0.79 J/cm^2^ and 1.1 J/cm^2^ respectively. The laser energy was less than the maximum permissible exposure, which is an important feature toward clinical applications of the eTC-PCT technique.

The brain images were reconstructed with “slice width” *W*_*T*_ = 10 ms for achieving the highest axial resolution for the camera frame rate of 104 Hz. For tumor imaging, because of the lower complexity of tissue, “slice width” of *W*_*T*_ = 100 ms was used for achieving higher signal quality. After extracting the slices, 3-D reconstruction of these data was achieved through ImageJ software using the Volume Viewer plugin. The ImageJ images (Fig. 3) are not on the same scale, and the software does not allow for associating quantitative values to image colors in the volume viewing format. 2-D slices were obtained by importing the eTC-PCT data into MATLAB. The brain and tumor image enhancement code was written and compiled in both Matlab and Python.

## Supplementary information

Supplementary information
