# Supplementary material for: Non-invasive in-vivo 3-D imaging of small animals using spatially filtered enhanced truncated-correlation photothermal coherence tomography
Source: Sci Rep. 2020 Aug 13;10:13743. doi: 10.1038/s41598-020-70815-3 (PMC7426848; doi:10.1038/s41598-020-70815-3)
Supplement: Supplementary file 1 — Supplementary information [file 41598_2020_70815_MOESM1_ESM.docx]

Non-Invasive *In-Vivo* 3-D Imaging of Small Animals Using Spatially Filtered Enhanced Truncated-Correlation Photothermal Coherence Tomography

Pantea Tavakolian^*^, Sohrab Roointan, Andreas Mandelis^*^

Center for Advanced Diffusion-Wave and Photoacoustic Technologies (CADIPT), Department of Mechanical and Industrial Engineering, University of Toronto, Toronto, M5S 3G8, Canada

*Corresponding author: [pantea.tavakolian@mail.utoronto.ca](mailto:pantea.tavakolian@mail.utoronto.ca); [mandelis@mie.utoronto.ca](mailto:mandelis@mie.utoronto.ca)

# Supplementary information

# Signal Processing theory of enhanced truncated correlation photothermal coherence tomography

In the enhanced TC-PCT technique, the function generator produces a Linear Frequency Modulated (LFM) pulsed chirp that controls the laser beam. The pulsed chirp excitation waveform is recorded by a data acquisition module for synthesizing the truncated reference chirp. The in-phase reference signal (R_0_), and its quadrature (R_90_) are synthesized from the recorded excitation chirp:

| $R_{0}(t)=\sum_{m=0}^{p} \int_{m}^{m+w_{T}} \delta\left[ t-\left( \frac{-\omega_{1}+\sqrt{\omega_{1}^{2}+2\pi r\left( 4m+1 \right)}}{2r} \right)-W_{T} \right]dW_{T}$ |  |
| --- | --- |
|  | (A1) |
| $R_{90}(t)=\sum_{m=0}^{p} \int_{m}^{m+w_{T}} \delta\left[ t-\left( \frac{-\omega_{1}+\sqrt{\omega_{1}^{2}+8m\pi r}}{2r} \right)-W_{T} \right]dW_{T}$ |  |
|  | (A2) |

Axial resolution in the 3-D TC-PCT image of the sample depends on the time gate ($W_{T}$) specified in Eqs. (A1) and (A2). The minimum value for the $W_{T}$ is limited by the camera frame rate, and the maximum is limited by the time difference between the last two pulses in the up-chirp waveform. Selecting a large time gate leads to a lower axial resolution, while it localizes more energy within the slice thickness, thus leading to higher SNR.

A schematic diagram of the reference waveforms, and a thermal response signal are shown in Figure A1. The reconstruction of the thermal relaxation signals is based on the match filtering technique well-known in radar science. The in-phase/quadrature reference signals are first shifted with different delay time (*d*), and different reference chirps (R_n,0_ and R_n,90,_ n=1,2,…,q) are generated, Figure A1. The technique cross-correlates the in-phase and the quadrature of the reference chirp (R_n,0_ and R_n,90_) with the photothermal images captured by the camera in a pixel-by-pixel format, Figure A2:

| ${CC}_{n;0/90}(t)=\int_{-\infty}^{\infty} R_{n; 0/90}^{*}(t+\tau) T_{chirp-pulse}\left( \tau\right)d\tau$ | (A3) |
| --- | --- |

Finally, the cross-correlation amplitude and phase can be calculated from

| $A_{CC, n}=\sqrt{{CC}_{n,0}^{2}+{CC}_{n,90}^{2}}$ | (A4) |
| --- | --- |

and

| $\emptyset_{cc, n}={tan}^{-1}({CC}_{n,90}/{CC}_{n,0}).$ | (A5) |
| --- | --- |

In our analysis, the amplitude peak value is measured from the cross-correlation amplitude, Eq. (A4). From the cross-correlation phase, Eq. (A5), the phase value at the amplitude peak time provides the phase of one pixel. To obtain truncated information from the full cross-correlation, the enhanced TC-PCT program searches for the amplitude peak based on the distance between the last two consecutive pulses in the full duration of the excitation signal waveform, and is based on the following formula for the applied delay to the reference signals:

| $Filtering width=Shortest pulse distance-(n-1)\times d$ | (A6) |
| --- | --- |

where $n$ is the slice number, and $d$ is the delay applied to the reference signal, shown in Figure A1.

For all pixels of the captured thermophotonic images, the truncation process provides tomographic slice eTC-PCT amplitude and phase planar images (tomograms) of an optically excited object. The time delay control represented by the time evolving filter $W_{T}$ imparts spatial coherence to the resulting tomograms, a feature that is absent from conventional diffusion-controlled depth integrated photothermal imaging. Amplitude and phase data for each pixel are measured from the cross-correlations using Eqs. (A4)and (A5).


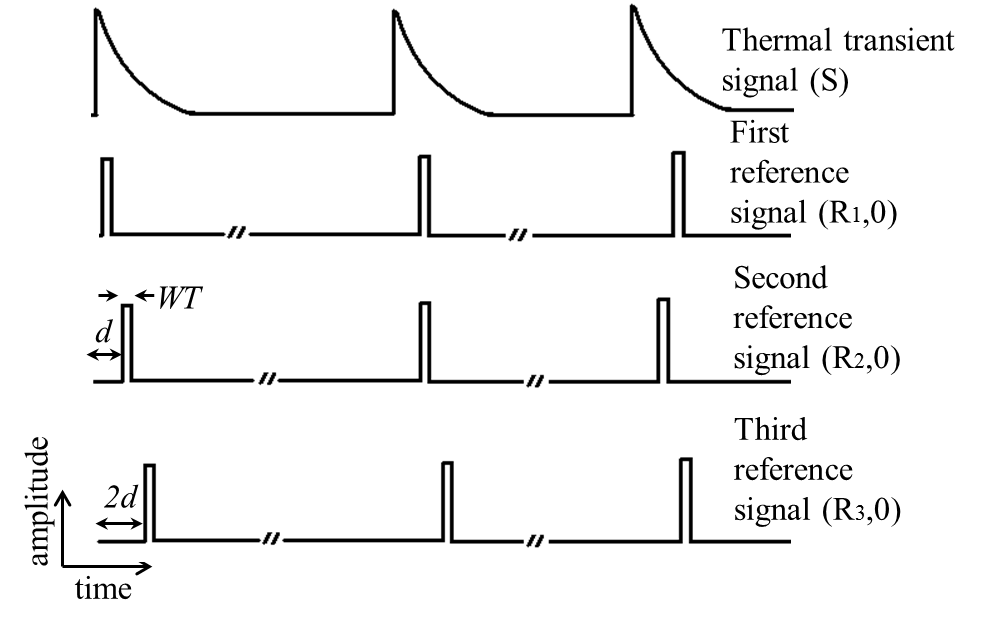


Figure A1.Schematic diagram of a thermal signal and reference signals. The reference signals are shifted by delay time of *d* and have pulse width of *WT,* which specifies the axial resolution of eTC-PCT images.

| (a) |  |
| --- | --- |
| (b) |  |
| (c) |  |
| Figure A2 eTC-PCT theoretical analysis of photothermal transient signals. (a) Thermal transient signal of a tumorous area in a mouse thigh . (b) An in-phase reference signal. (c) Cross-correlation amplitudes of transients with in-phase reference signal. The *x* axis here is time that can be measured in seconds by dividing the time point over the 104 (camera frame rate). | |
